# Nonunion of humeral medial condyle fracture caused by excessive functional exercise: a case report and review of the literature

**DOI:** 10.1186/s12891-022-05932-3

**Published:** 2022-11-08

**Authors:** Liu Yang, Feng Xia, Jia-chao Guo, Xiao-lin Wang, Jin-peng He, Jing-fan Shao

**Affiliations:** 1 Department of Pediatric Surgery, Tongji Hospital of Tongji Medical College, Huazhong University of Science and Technology, Wuhan, Hubei China; 2 Department of Hepatic Surgery, Tongji Hospital of Tongji Medical College, Huazhong University of Science and Technology, Wuhan, Hubei China

**Keywords:** Functional exercise, Medial humeral condyle fracture, Children, Screw fixation, Osteotomy

## Abstract

**Background:**

Medial epicondyle fractures are one of the more common humerus fractures, but humeral medial condyle fracture (HMCF) is rare. Nonunion of medial humeral condyle fractures due to functional exercise is less common.

**Case presentation:**

We report a 5-year-old patient with a nonunion HMCF due to excessive functional exercise, who bruised the elbow 1 year ago and had no positive findings on all imaging studies. On this physical examination, there was a snapping and palpable lump in the elbow joint during movement, but the patient did not feel any discomfort and the range of motion of the joint was normal. X rays and computed tomography (CT) showed that the left HMCF was discontinuous, the broken ends were dislocated, and the joint alignment was poor. Open reduction (OR) and screw fixation was used during the operation, and the patient recovered well at 3-month follow-up.

**Conclusions:**

The rarity and low radiographic appearance of displaced HMCF are easily overlooked and can eventually lead to nonunion HMCF, especially when radiographically difficult to visualize before age 5 years. Therefore, regardless of whether there are signs or imaging abnormalities in the growth process of adolescents, they should be vigilant, shorten the time interval for re-examination, and early detection and timely treatment can avoid some complications caused by this.

## Background

Medial humeral condyle fractures(HMCFs) are very rare Salter-Harris IV fracture [1]. Bensahel et al. reported that HMCF occurred in less than 2 percent of elbow fractures in adolescents, with a high incidence between the ages of 8 and 12 years [2]. There are two generally accepted mechanisms of injury, one is a direct blow to the posterior proximal ulna during elbow flexion, separating the medial condyle. The second mechanism is the fracture of the medial condyle when it is pulled by muscles and tendons during a fall [3]. It is worth adding that Namba J et al. reported a 15-year-old patient with HMCF, and the authors demonstrated by means of 3D reconstruction that wedge force can directly split the trochlea [4].

The ossification center of the trochlear generally begins at age 7 years, and the ossification center of the medial epicondyle generally begins between the ages of 5 and 9 years [5–7]. In children over 8 years old, most of the trochlear and medial epicondyle have begun to ossify. Coupled with a clear history of elbow injury and imaging features (although not obvious, but traceable), the probability of being overlooked is greatly reduced. HMCF that occur under the age of 5 years, the medial condyle and trochlear are both cartilage, the imaging signs are basically absent, the diagnosis is very difficult, it is easy to delay the disease, and it evolves into a nonunion HMCF. This leads to late complications, such as limited elbow motion, cubitus varus/valgus deformity, delayed ulnar nerve palsy and so on [5].

HMCF appear to be mirror images of lateral humeral condyle fractures in most of the literature reported by investigators and contribute to the proposed management of lateral humeral condyle fractures[8]. When HMCFs are initially diagnosed, OR and internal fixation usually achieves the desired effect [9]. However, for neglected HMCF, most orthopedic surgeons choose OR and internal fixation or osteotomy according to the adolescent's physical signs and personal habits [10–12]. Papavasilion et al. and Varma et al. treated nonunion HMCF by corrective supracondylar osteotomy [13, 14]. Satisfactory results were also achieved with OR and internal fixation by Soon KS et al. [12]. The choice of surgical approach has been controversial. It is difficult to compare the pros and cons of the two procedures because of the small number of cases and the large age range (the child has a steep growth curve).

There are not many reports about the nonunion HMCF in children, but the neglected HMCF is caused by the fact that the doctor at the first diagnosis requires the patient to perform early functional activities, and the parents ignore the symptoms of the patient's elbow pain and force functional exercises. No relevant reports have been found before. Here, we report a case of a 5-year-old boy who underwent premature functional exercise due to the negligence of both doctors and parents at the initial admission, which eventually led to the nonunion HMCF. We chose to open it. Reduction and internal fixation, followed up after 3 months, no pain, no joint snapping. And we also provide a literature review on the surgical methods, prognosis, and complications of nonunion HMCF. In addition, we discuss the need for surgery for nonunion HMCF based on published studies.

## Case presentation

In February 2022, a 5-year-old male patient was admitted to the Department of Pediatric Surgery of Tongji Hospital, Hubei Province, China. The chief complaint was that he could hear and snap when the left elbow joint was flexed, and a hard mass could be palpated medially. The child suffered a bruised left elbow while playing in the playground 1 year ago, and then developed upper extremity pain with limited mobility, normal extension, and range of motion (ROM) of 60°. The CT plain scan and three-dimensional reconstruction examination showed that there were no obvious signs of dislocation fracture and dislocation of the left elbow joint. The doctor who received the initial consultation did not pay attention to the signs of the elbow, and did nothing to deal with it, but only recommended to start functional exercise after two weeks of rest.

During this period, the patient's elbow pain did not increase over time. But the parents strictly followed the doctor's warning. Finally, after 20 days of functional exercise on the left elbow, there is a snapping sound at the joint when flexing. After 1 month of elbow activity, the family suddenly found that there was a 2*1 cm mass above the olecranon of the left elbow, which was hard and could not be pushed. Then the parents asked the doctor who received the first visit, but the doctor still did not pay attention to it, thinking that the bulging mass was formed after muscle contracture and would gradually disappear. After 1 year, the parents found that the patient's elbow was unstable, prolapsed with a small medial pressure, and the patient resisted pressing on the inside of the left elbow. Then came to our hospital for treatment.

**Fig. 1:**
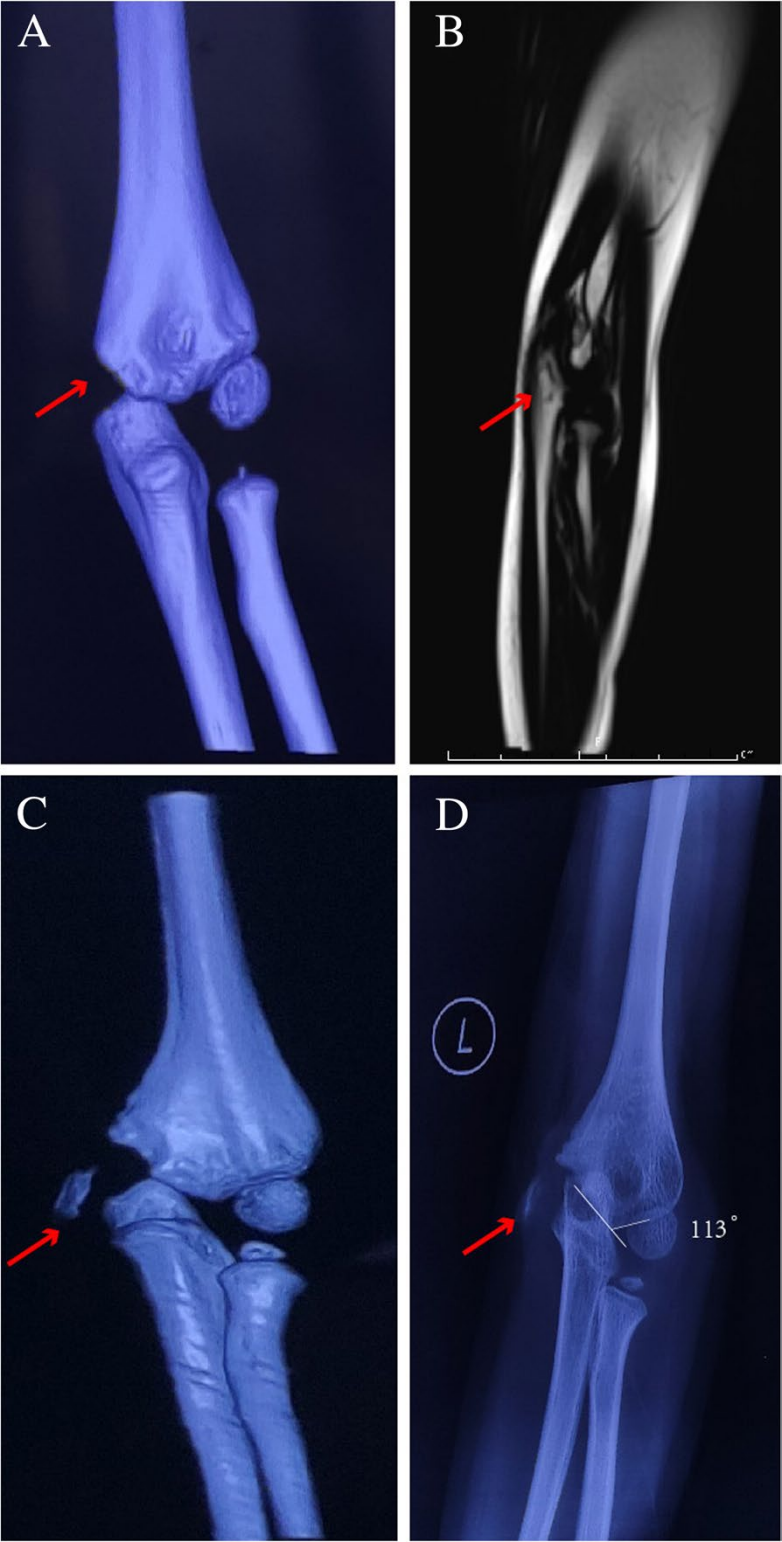
Imaging features of patients. **A** 3D-CT of the elbow 1 year ago, no abnormality was seen. **B** This MRI showed poor joint alignment and joint effusion. **C** Falling debris is clearly visible in the 3D reconstructed image. **D** X-ray examination can clearly show that the bone cortex is discontinuous and the broken ends are dislocated

This X rays and computed tomography imaging with three-dimensional reconstruction(3D-CT) showed that the left medial epicondyle was irregular in shape, the edge of the humerus was photo-sclerotic, and the humerus-ulnar correspondence was not good, so the left medial condyle was considered nonunion. There was no previous family history, and biochemical findings were unremarkable; on physical examination, there was joint snapping during flexion of the left elbow. The range of motion of the joints was normal, and the flexion and extension were not restricted, but the joints were unstable, and the blood supply and activities of the fingers were fine. The magnetic resonance imaging (MRI) examination in our hospital showed that the left medial condyle of the humerus was discontinuous, the broken end was dislocated, and the elbow joint cavity was effusion. We recommend surgical treatment (Fig. 1A-D).

During the operation, an incision was made with the medial condyle of the humerus as the center, the flap was opened to both sides, and the subcutaneous tissue was separated. Behind the medial muscle septum. The ulnar nerve was isolated and protected with a rubber drainage tube to avoid intraoperative injury. Muscles were then separated, and the brachialis and biceps and triceps were separated along the medial epicondyle of the humerus to expose the medial distal humerus, the posteromedial aspect of the elbow joint, and the ulnar portion of the olecranon notch. Clear and clear pale yellow synovial fluid was seen flowing out, and the free bone fragment was located on the ulna and had been fully rotated. An obvious defect was seen at the medial condyle of the left humerus, and the wound had obvious fibrotic tissue. A 2.0*1.5 cm free bone fragment (mostly cartilage components) can be seen above the olecranon of the ulna. There is a common flexor tendon attachment on one side of the bone fragment. In fractures of the distal humerus, a bone pluck is used to clear the surface of fibrotic tissue to create a fresh wound.

**Fig. 2:**
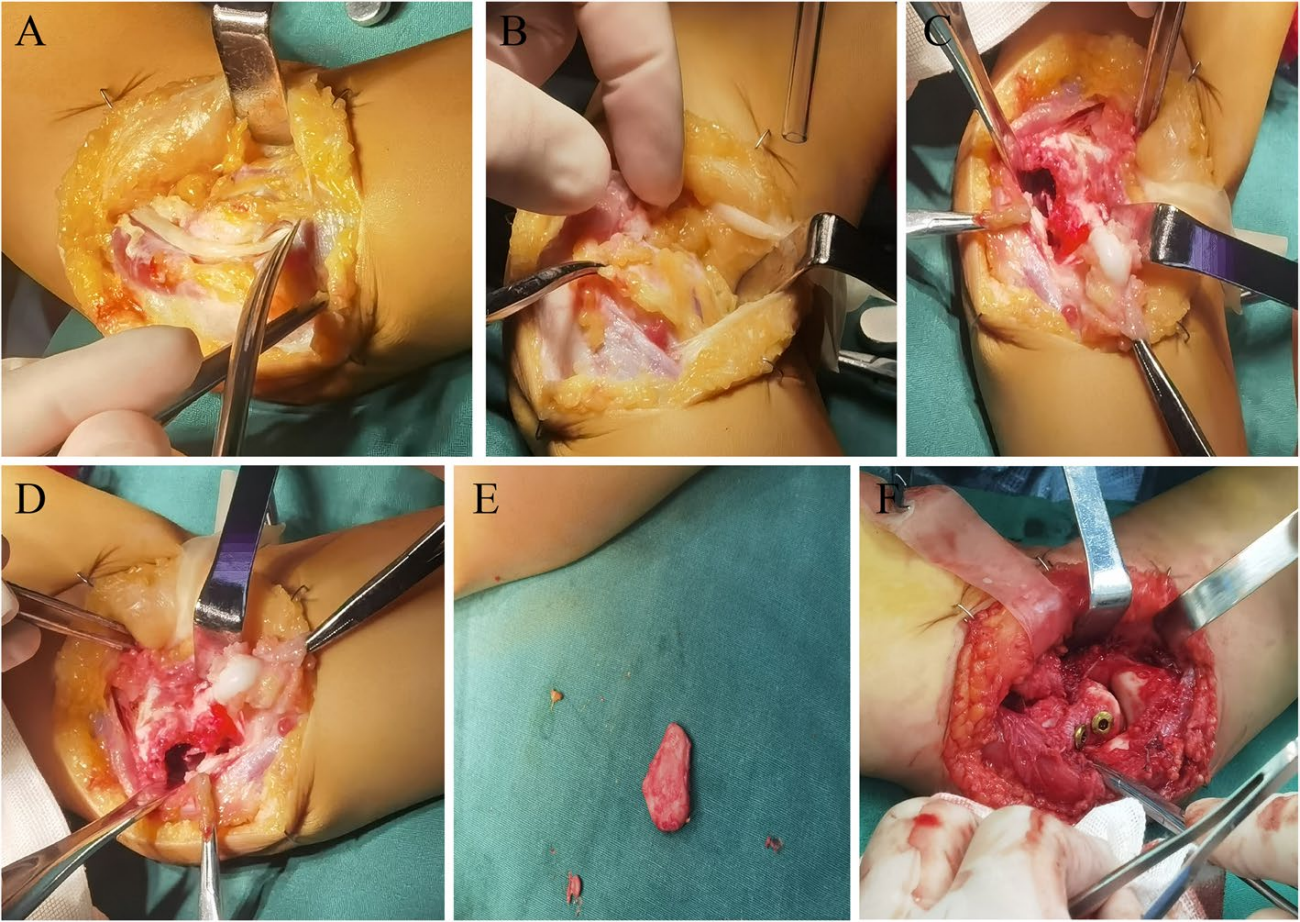
Internal fixation of HMCF. **A** Find the ulnar nerve to separate and protect it. **B** Enter the joint cavity to find the fracture end and bone fragments. **C**-**D** The bone fragments and the medial condyle fragment can be seen after ossification. **E** The separated bone fragments, 3 × 2x1cm size. **F** Two hollow metal screws fixed

**Fig. 3:**
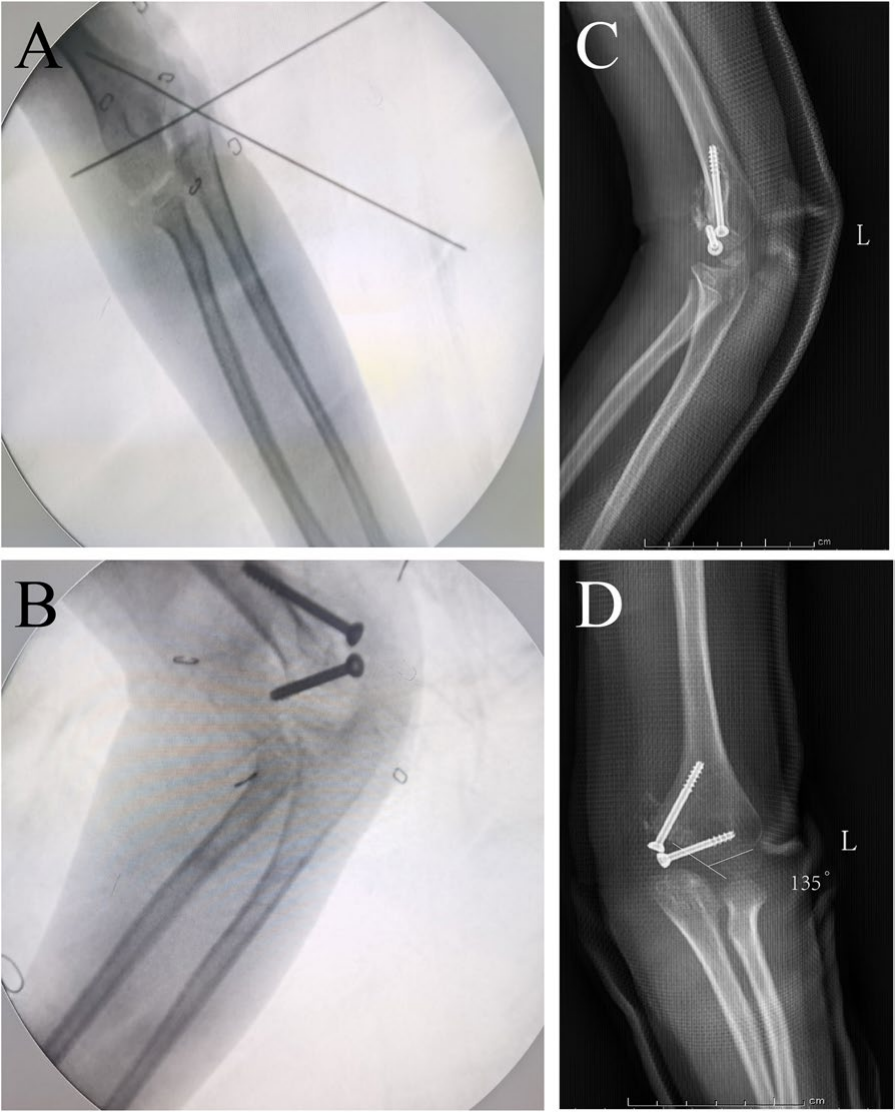
Intraoperative and postoperative X rays. **A** Using Kirschner wire for positioning. **B** Using metal screw fixation. **C**-**D** Postoperative X-ray appearance after cast immobilization

Due to the long time, the common flexor attached to the fragment retracted, and the free bone fragment could not be pulled back to the defect during the operation, so the muscle attached to the free bone fragment was cut off. The bone fragments were placed in the original defect, but the surface and shape of the fragments and the defect had occurred after positioning with two smooth Kirschner wires as guide wires. Two metal cannulated screws were fixed, and then the collateral ligament was reconstructed. Since the original ulnar nerve groove was missing, the nerve was moved forward, and the incision was sutured. The arm was cast in place for 4 weeks (Fig. 2A-F). X rays were reviewed postoperatively, and bone fragment reduction was seen (Fig. 3A-D). The patient presented with mild ulnar nerve palsy on the one day after operation, which subsequently recovered normal. At the 10-week follow-up, the patient had normal range of motion and full range of motion in the left elbow. At present, patients do functional exercise to avoid muscle atrophy.

## Discussion

Fractures Medial epicondyle of the humerus differ from HMCF in that the former is an extra-articular fracture and the latter is an intra-articular fracture involving the epiphysis [1, 2]. HMCF typically involve only one of the them known mechanisms of injury. However, in this case, there were both reasons for the nonunion HMCF. The first was the direct impact of violence on the medial condyle of the humerus during play, resulting in the fracture of the medial condyle, which was not dislocated at this time. However, because the patient's trochlear ossification center has not yet appeared, the X rays did not reveal the fracture line. Premature elbow movement after two weeks was the culprit for rotational displacement of the fracture.

When the secondary ossification centers of the trochlea and the medial epicondyle are clear, the fracture of HMCF is not easy to be missed or misdiagnosed. However, children's pulleys usually do not become ossified until the age of 9, and studies have shown that children under 6 years of age have a higher risk of misdiagnosis [2, 5, 6]. It is difficult to diagnose on X rays, even on CT. In this case, 3D-CT was also not found [15]. HMCF are through the epiphysis, when ignored, have a high probability of nonunion, cubitus varus/valgus, and joint stiffness [1, 11]. HMCF are less likely to be missed or misdiagnosed when both the trochlear and medial epicondyle secondary ossification centers are clearly defined. Some studies have shown that MRI is the best method to diagnose the fracture of medial condyle of humerus which has not appeared in the ossification center of trochlea [16]. But MRI are expensive, time-consuming, and children are often reluctant to cooperate because of their noise. Even children need to be sedated before it can be done, and parents often refuse MRI.

In type III dislocated HMCF, the diagnosis is easy and the indications for surgery are clear. However, since the fracture of the cartilage part is difficult to visualize, the diagnosis of a fracture that is not displaced is very difficult. In this study, initial imaging and elbow signs were inconspicuous and easily overlooked. It is worth noting that when a lump appears in the elbow after premature exercise, the fracture fragment has actually been rotated and displaced. But it still went unnoticed by doctors. Primary doctors are unfamiliar with rare diseases and rely too much on imaging techniques. Negative imaging results cannot rule out the diagnosis of medial humeral condyle fracture, and caution should be exercised during physical examination of the patient. Further evaluation by senior physician.

If necessary, polymer plaster can be used for immobilization, 3D-CT should be reviewed after two weeks. If no further diagnosis can be made, regular follow-up and timely follow-up can be performed. can be detected early [17]. A part of the reported cases is due to the falling or obvious displacement of the fracture fragments, which can be detected and treated in time by imaging examination.Sugiura et al. reported a case of cubitus varus malunion one year after conservative treatment of a medial humeral condyle fracture [10]. Reports of such cases are rare. Our patient had no elbow deformity one year later, but had developed underlying manifestations of joint instability. Surgery becomes necessary, and although the joints move normally now, there is a high possibility of deformity with age.

The diagnosis of fracture of medial condyle of humerus in childhood is still difficult, including the following points: (1) The limitation of MRI for children, which is expensive, time-consuming, noisy and uncooperative; (2) Primary health care resources are limited, and some local medical institutions are not equipped with nuclear magnetic resonance imagers and CT scanners; (3) The grass-roots doctors are inexperienced and do not understand the development and change process of children's ossification center.

According to the literature, ultrasound supports the role of dorsal fat pad sign (equivalent to joint effusion) in olecranon fossa in children under 12 years of age with elbow injury. The symptom of absence of fat pad can rule out 98.2% of fracture (negative predictive value) [18–22]. In 1998, de Maesener proved that the sensitivity of ultrasonography in detecting fat pad signs was higher than that of X-ray, and only MRI was more accurate than ultrasound [21]. Therefore, considering the cost-effectiveness and limited local health system conditions, ultrasound can replace X-ray as the preferred examination method for children with elbow injury under the age of 12. Early diagnosis of HMCF can be made by: (1)History of elbow injury, (2)Imaging examination, ultrasound and MRI are mainly used in children under 12 years old, X-ray and CT are used in children over 12 years old.

Neglected HMCF is rare in children and account for a disproportionate number of rare HMCF. So far, only 12 cases of nonunion HMCF due to early neglect and delayed treatment due to various factors have been reported in the review of the English-language literature. In the 12 cases, the mean age of injury was 6 years, and all 12 patients were ignored because the X rays showed no abnormality or the elbow signs were not obvious. Four patients underwent OR Kirschner wire fixation, 3 patients underwent OR metal screw fixation, 2 patients underwent closed osteotomy, and the remaining 2 patients underwent condylar fixation Upper osteotomy. 4 patients who underwent osteotomy both developed severe cubitus varus, and OR and internal fixation could be performed directly when there was no cubitus varus or mild cubitus varus did not affect joint function (Table 1). In our case, the patient underwent OR with metal screw fixation because the patient did not have cubitus varus.

**Table 1:** Various parameters and surgical methods of 12 patients with nonunion HMCF

Author	Case no	Age	Sex	Side	Type	Injured age	Delay to treatment	Elbow deformity	Surgery	Outcome
Su HC [11]	3	21	F	R	?	9	11 years	Cubitus varus deformity	OR/screw	Good
		18	M	R	I	5	13 years	Cubitus varus deformity	Supracondylar dome osteotomy (SDO)	Good
		11	F	L	?	6	5 years	Cubitus varus deformity	Supracondylar dome osteotomy (SDO)	Good
Sugiura H [10]	1	10	M	R	I	4	6 years	Cubitus varus deformity	Closing wedge osteotomy	Good
Fernandez FF [9]	3	7	M	R	III	6	1 years	None	OR/screw	Normal
		6	M	R	III	6	5 months	None	OR/K-wire	Normal
		6	M	R	III	6	5 days	None	OR/K-wire	Good
Ryu K [14]	1	14	M	R	?	3	11 years	None	OR/screw	Good
Song KS [12]	1	10	M	L	I	4	6 years	Cubitus varus deformity	OR/K-wire	Good
Varma BP [8]	2	16	M	L	I	10	6 years	Cubitus varus deformity	OR/K-wire	Good
		7	F	L	I	7	6 weeks	Cubitus varus deformity	Closing wedge osteotomy	Good

## Conclusions

when adolescent with history of elbow fall and no fracture is found on imaging, the primary doctor should be cautious, inquire about the injury history in detail, perform MRI if necessary, or perform additional evaluation, such as under anesthesia. Arthrography. Cast immobilization should also be maintained for 4 weeks to shorten the review interval. It can effectively avoid the occurrence of neglected medial humeral condyle fractures.

## Data Availability

The datasets used and analyzed during the current study are available from the corresponding author on reasonable request.

## Abbreviations

HMCF: Humeral medial condyle fracture
CT: Computed tomography
OR: Open reduction
ROM: Range of motion
MRI: Magnetic resonance imaging
3D-CT: Three-dimensional reconstruction
